# Information Dissemination of Public Health Emergency on Social Networks and Intelligent Computation

**DOI:** 10.1155/2015/181038

**Published:** 2015-11-02

**Authors:** Hongzhi Hu, Huajuan Mao, Xiaohua Hu, Feng Hu, Xuemin Sun, Zaiping Jing, Yunsuo Duan

**Affiliations:** ^1^School of Management, Fudan University, Shanghai 200433, China; ^2^Department of Vascular Surgery, Changhai Hospital, Second Military Medical University, Shanghai 200433, China; ^3^Department of Information, Changhai Hospital, Second Military Medical University, Shanghai 200433, China; ^4^Department of Respiratory Medicine, Shanghai Tongren Hospital, Shanghai Jiao Tong University, Shanghai 200336, China; ^5^Department of General Surgery, Tongji Hospital Affiliated to Tongji University, Shanghai 200065, China; ^6^Psychiatry Department, Columbia University/New York State Psychiatric Institute, New York City, NY 10032, USA

## Abstract

Due to the extensive social influence, public health emergency has attracted great attention in today's society. The booming social network is becoming a main information dissemination platform of those events and caused high concerns in emergency management, among which a good prediction of information dissemination in social networks is necessary for estimating the event's social impacts and making a proper strategy. However, information dissemination is largely affected by complex interactive activities and group behaviors in social network; the existing methods and models are limited to achieve a satisfactory prediction result due to the open changeable social connections and uncertain information processing behaviors. ACP (artificial societies, computational experiments, and parallel execution) provides an effective way to simulate the real situation. In order to obtain better information dissemination prediction in social networks, this paper proposes an intelligent computation method under the framework of TDF (Theory-Data-Feedback) based on ACP simulation system which was successfully applied to the analysis of A (H1N1) Flu emergency.

## 1. Introduction

Public health emergency refers to a sudden event which may cause serious damage to the social public health such as major infectious disease, mass unknown illnesses, and major food and occupational poisoning. In today's society, public health emergencies, such SARS in 2003, Bird Flu in 2006, A (H1N1) Flu in 2009, and EBHF in 2014, have triggered extensive social influence by the dissemination of related information.

In order to get a comprehensive understanding of unconventional emergencies which possibly bring serious impacts on the country and society and improve the government's coping capacity, a major research plan was launched by the NSFC (Natural Science Foundation of China) in 2009 and organized a large interdisciplinary research community with more than 300 scientists, technicians, and engineers. As an important part of the above research plan, our work is focused on the information dissemination and computation associated with public health emergency.

Social networks have become an open complex giant system that connected more than two-fifths of the world populations and acted as the main platform of information dissemination in public health emergency, which can easily cause the spread of false facts or rumors and bring about a serious panic without efficient management [1, 2]. Therefore, the prediction of information dissemination in social networks is necessary for estimating the social impacts and making a proper strategy in the management of public health emergency.

In the past decades, numerous research works have been devoted to social networks [3–7]. Scholars conducted the technologies of content analysis and topic detection to study the information spread path as well as its key nodes [4, 5] and furthermore probed into the topological structure and organizational mechanism of the spread network [6–8]. It has been realized that information dissemination in social networks is affected not only by the variable network's structure and dynamically changing spread paths, but also by the complex interactive activities and group behaviors in social communities [7, 8]. Therefore, statistical models such as time regression or estimators based on the traditional machine learning technologies are difficult to achieve good prediction results [6, 9].

In recent years, in the view of complex adaptive systems, researchers put forward many models to describe the information dissemination characteristics in social networks, such as “the small world” model, information diffusion model of dynamics, and infectious disease diffusion model like SIS (Susceptible, Infected, and Susceptible) [10, 11]. Those research findings described the behavioral mechanism and theoretical characteristics in the process of information dissemination. However, there are some barriers to be applied in the prediction of real situation [9].

On the other hand, some researches were focused on data mining and established the predictive model based on the historical data and existing cases. There are also some limitations due to the dynamic changing social connections in cyber space and the uncertain processing behaviors which are largely affected by the cognition and reactions of members in social networks [6]. Considering these complex characteristics and factors, an effective computation method aiming at achieving a better prediction result of information dissemination needs to be explored.

## 2. Complex Characteristics of Information Dissemination in Social Networks

### 2.1. Network Structure of Information Dissemination

The online forum, Internet micro-blog, instant messaging platforms, mobile network, and various communication terminals consist of a highly complex system [12, 13] for people to share information with others and express their opinions to the public. The increasing scale of information dissemination makes existing mathematical model difficult to describe the real situations and simulate the dynamic process adequately.

Once an emergency event takes place, the information dissemination will usually experience a life cycle of five stages: incubation stage, outbreak stage, diffusion stage, decaying stage, and aftermath stage [8]. According to the existing research findings [6], the information dissemination in social networks appears in the forms of two kinds of patterns: the public pattern such as BBS and micro-blog, and the small world pattern such as Wechat and QQ. The network structure of information dissemination in public pattern is an open and unstable structure with a lot of uncertainty. However, the structure in the small world pattern is relatively stable but has higher efficiency and easily leads to group behaviors because it is usually based on the familiar members and a vocal media system [14].

**Figure 1:**
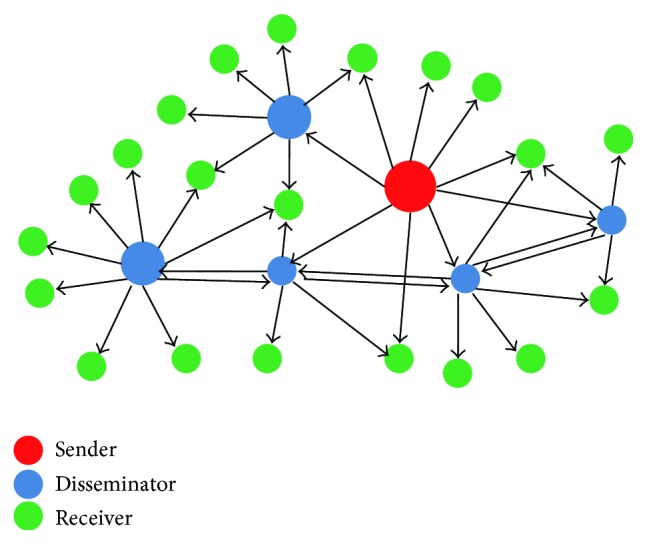
Participants of information dissemination in social networks.

In public health emergency, information dissemination in social network involves three roles of the participants [15]: information senders, information disseminators, and information receivers, as shown in Figure 1.

**Figure 2:**
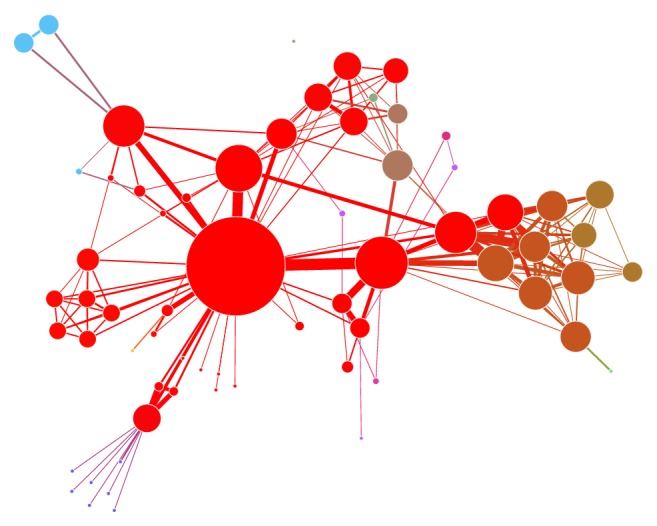
The leading nodes and paths of information dissemination in social network.

However, the roles of participants are often changing by many factors during the process of dissemination with different kinds of events, such as interest and attention, the level of activity, and the time to catch relevant information. This will lead to dynamic changes of spread paths and network structure under different events. For example, Figure 2 shows its main leading nodes and paths of information dissemination in a social network which are expressed in different size and colors. The larger red nodes in the figure are the leading nodes, and the thicker links of each node are the main paths.

Different from the inflexible structure in electronic circuit, the connections in social networks are actually flexible and random. It means that the features of network structure are dependent on the dynamic strength among different nodes. For convenience, we define the expressions as follows.

The connections in social networks can be regarded as a graph *G*, and

(1) $$\begin{array}{c} G=\left\{V,E\right\}, \end{array}$$

where *V* is the collection of nodes and *E* is the collection of connections among the nodes:

(2) $$\begin{array}{c} V=\left\{v_{i}\right\},\;i=1,2,3,\ldots ,M,\\ E=\left\{e_{j}\right\},\;j=1,2,3,\ldots ,N. \end{array}$$

In the above, *k*_*i*_ is the number of active communication on connection *e*_*j*_ in a certain period *t*; then *e*_*j*_ is an effective connection if *k* > 0, or an ineffective connection if *k* = 0.

**Figure 3:**
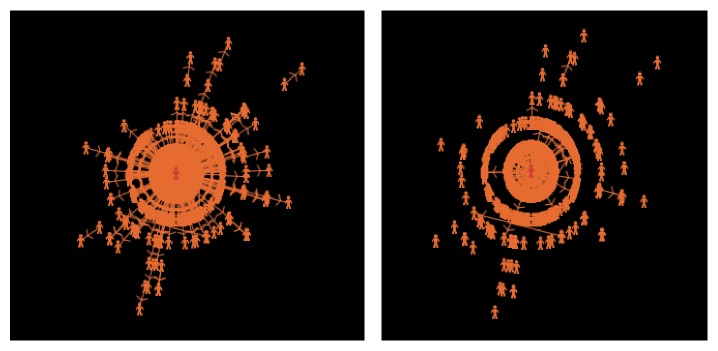
Dynamic structure of a micro-blog network when the spreading threshold varies from 0.3 to 0.5.

Figure 3 shows the dynamic structure of a micro-blog network when the spreading threshold varies from 0.3 to 0.5 [15]. It can be observed that the network structure is changing with time by different connection strengths; some connections may disappear but those strong connections still exist.

However, in the prediction of information dissemination in social networks, we should consider the complicated characteristics of its based network structure [6]. We will discuss this in the following section.

### 2.2. Cognitive Psychology and Group Behaviors

It has been verified by many real cases and empirical studies that information dissemination in social networks has close relationship with the participants' psychological reactions and their behaviors in particular situational context [6–8, 16, 17].

The process of information dissemination may be affected by many factors, among which the emotions and behaviors of participants have direct impacts on the development of public opinions. In social network, users can release and disseminate all types of information with real names or anonymous way, while the validities or authenticity of the information may be difficult to determine at first glance [18]. The information dissemination is thus beyond the technological control and subject to individual attitude, emotion, and behavior and largely depends on the trust of network relations and human cognitive process under specific situations. Furthermore, the individual cognitive nature of attitude, emotion, and behavior will easily result in the group behaviors in social networks.

Cognitive theory of emotion considers that emotion is affected by three factors: environmental events, physical condition, and cognitive process, among which cognitive process is the decisive factor [19]; the information processing especially elaborates on the content of the received information. According to limited cognitive resources, individuals usually identify and process information in terms of selective attention in cognitive processing [20].

In addition to the information content, people also consider the factors of information itself, for example, source credibility and information length. Some research shows that the social and cultural backgrounds and emotions of information disseminators will affect their cognitive process significantly, which is obvious in the Internet public opinions. For instance, some information will be largely reproduced, spread, and reposted in a very short time and then lead to large-scale riots and even affect daily life. In the above process, cognitive results and emotional reactions exhibited by groups are the core elements that lead directly to their coping strategies and behaviors; these mechanisms have been systematically studied by Bagozzi and Dholakia [21], Wheeless and Grotz [22], and Barnes and Olson [23] from the aspects of self-disclosure, opinion leaders, and opinion followers on social networks.

Recent researches of cognitive psychology and group behaviors in social networks have developed into a new interdisciplinary field which was called Cyber Psychology and defined as “understanding people how to react and behave within cyberspace” by Dr. Suler in his hypertext book* The Psychology of Cyberspace* [24]. The progress in social neuroscience [25] has provided new aspects for achieving a better study in this field. Remarkably, modern technology of functional magnetic resonance imaging (abbreviate to f-MRI) can directly track reactions involved with all types of situational information stimuli in cognitive process [26, 27], and ERPs (short for Event-related Potentials) can get high resolution in dynamic process which has been used to study sensitive reactions [28]. These will help to explore the brain mechanism of cognition in information dissemination.

Based on new theory and advanced experimental technologies, researchers have found complicated factors related to cognition and group behaviors which may affect the information dissemination of public health emergency in social networks, such as attention and interest on the events, psychological characteristics of people with different regions, cultural background, and specific situations context. However, the existing findings are mostly obtained by questionnaire surveys or experimental observations which may easily lead to the incomplete results due to the limitations of investigated samples or particular experimental cases. So, find a systemic way that is needed in applying in real environment [6].

According to our previous research [6], we found that the dissemination behaviors for an information receiver are related to the factors as in the following formula:

(3) $$\begin{array}{c} B_{t+1}=f\left(C,A_{t},E_{t},B_{t}\right), \end{array}$$

where *B*_*t*+1_ is the dissemination behavior of an information receiver at time *t* + 1. *C* denotes the event's content; *A*_*t*_, *E*_*t*_, and *B*_*t*_ are the states of the information receiver's attitude, emotion, and his current behavior, respectively, at time *t*.

However, the quantitative relationship in formula (3) cannot be calculated accurately only by a simple mathematical formula. Some knowledge such as rules and behavioral characteristics of the information receiver are required [6]. In order to get an approximate estimation in the next time period, Kalman Filtering is a better choice shown in the following formula:

(4) $$\begin{array}{c} B_{t+1}\left(t\;|\;t-1\right)=LB_{t}\left(t-1\;|\;t-1\right)+MU\left(t\right). \end{array}$$

Information dissemination in social networks also pertains to the time period of occurrence, spread channels, type and quantity, and other dynamic circumstances. Moreover, the way of its dissemination is complicated, and the relations between them are complex [17]. Each participant may be regarded as a dissemination node, and it is not only the receiver but also a sender. The information dissemination through the Internet concerning the event is a complex mode and is featured to be divergent and fast. Daily interpersonal communications and telecommunication communications have been fused into information dissemination in whole social networks and constitute complex patterns of dissemination.

## 3. Intelligent Computation Method Based on TDF Framework

### 3.1. Limitations of Existing Methods

Unlike deep and wide research that was carried on epidemic spreading, quantitative research on the social networks' information dissemination has been relatively limited, and, furthermore, the existing methods do not sufficiently take into account the effects of social cognition, group behaviors, multichannel dissemination, and other characteristics or just make some macroscopic and statistical descriptive models. For the above reasons, we recognize that in practical applications, theoretical tools and modeling methods are difficult to adequately describe and accurately predict the information dissemination in social network. The main reason leads to the abovementioned problems being that the dissemination is affected by too many complex factors in dynamic changing environment due to cognitive psychology, emotional reactions, and behavioral intention of the network users which may result in uncontrollable processing behaviors and will continually produce new stimulus to other receivers. Under these circumstances, we find that it is not enough to solve this problem if we only rely on various theoretical models to describe basic features or artificial simulation systems derived from the historical data mining in such an open and uncertain environment.

On the whole, the existing methods have the following shortcomings: (1) It is difficult to apply a statistical model to predict actual situations in open and dynamic environment with macroscopic description based on sample data. (2) Some theoretical models can reflect the mechanism and some basic rules under an ideal condition, but there are huge variations as to actual situation in the real world. (3) Technologies of big data mining can provide valuable analysis and parameters for those models, but the future uncertainties may not accord with the changing tendency obtained by historical data. So, it needs further exploration to find a more effective method to deal with such complicated problem.

### 3.2. ACP Simulation System

Information dissemination in social networks involves an interdisciplinary study which covers psychology, sociology, mathematics, management science, and computer engineering. Such work is difficult to be clearly expressed just by mathematical computation formulas but should utilize the unstructured knowledge such as a variety of rules, empirical data, and cases. It is expected to exploit new ideas and methods from the integrated perspective of a real application environment.

**Figure 4:**
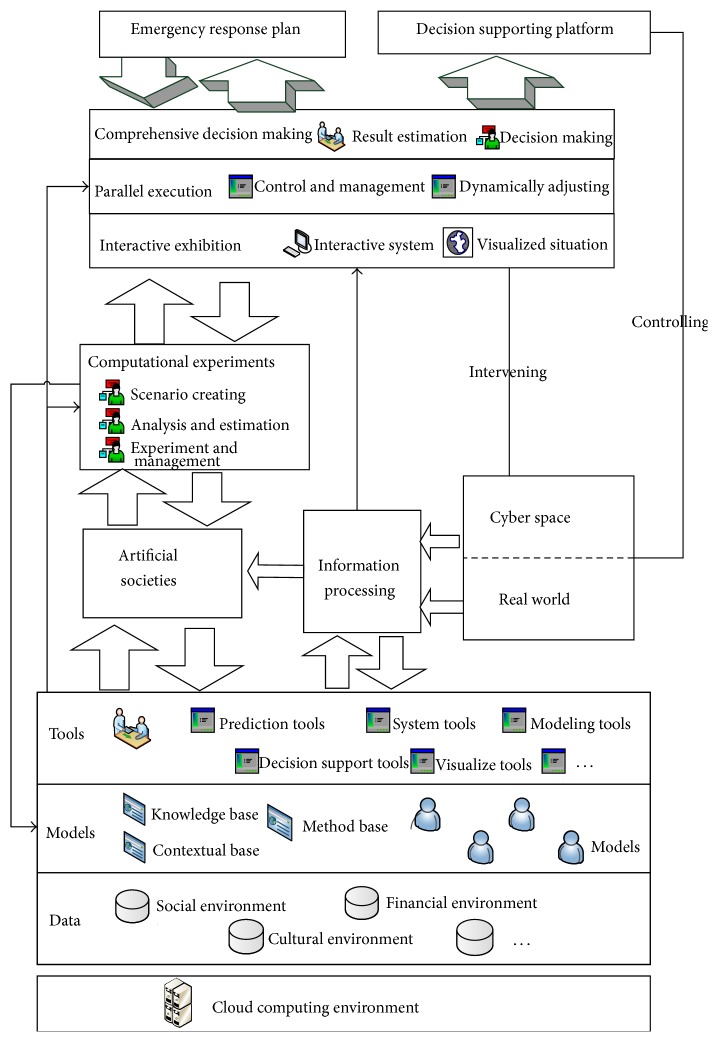
ACP simulation.

For the purpose of exploring how to offer comprehensive solution to the scientific problems of complex social and economic system, Wang from Institute of Automation, Chinese Academy of Sciences, firstly proposed a parallel computational theory to solve management and control issues in complex system and put forth social computing method that combined the artificial society with computational experiments and parallel execution, which is called ACP (artificial societies, computational experiments, and the parallel execution) [29]. This method can deal with the difficult problems such as Cyber Psychological computation and make it computable; Figure 4 shows the ACP simulation system which has been successfully applied to the dynamic analysis in the national emergency management and responses of China [6, 30].

ACP simulation system provides an effective way to solve the above problem and new clue to further experimental research. By using this, complicated problems which are not easy to be accurately predicted, difficult to precisely model, and unable to repeat experiments in real social system all can be addressed. In this computing environment, the real-time system continuously exchanges data and synchronously modifies the artificial social context in the light of the feedback data. Through iterative interaction and persistent approximation, it can help to realize the parallel computation between artificial society and real society and finally achieve the goal of dynamic optimization management and control. The ACP method can also serve as a bridge between social problem and computing technology and break the dilemma of interdisciplinary study between social and computation science. The method also has important significance in virtual community information dissemination from the perspectives of fundamental theory, experimental methods, key technology, and practical applications.

Information dissemination in social networks has very close relationship with psychological and emotional behavior in particular situational context, as well as its temporal-evolution-characteristics and complex spreading mechanism. In the face of all uncertainties of future context, in order to gain relevant experiential knowledge and important data parameters, further study should focus on cognitive mechanism of network information, trend predictions, and reaction rules. Only through deep experimental observations, instant social psychological research in addition to large exact cases will be able to make more accurate analysis and evaluation on the information dissemination rules, evolution characteristics, and developing trends.

### 3.3. Intelligent Computation Based on TDF Framework

In order to get a better prediction of information dissemination in social networks, Professor Dai proposed a new TDF (Theory-Data-Feedback) framework to deal with the modeling problem [9]. His framework absorbed the merits of the mechanism model, data model, and social psychological feedback model interacted with instant online survey data and formed a new systematic analysis and modeling method. We extended that framework to be applied in ACP simulation system for analyzing the information dissemination of public health emergency as follows.

The mechanism model includes the basic laws of things change, existing research results, and related prior knowledge on similar problems, such as network information spreading mechanism and evolution characteristics. It lays foundation for normal operation of the whole model. In this part, information dissemination mechanism needs comprehensive analysis to determine the range of data acquisition in “input” part of the model, which is of great importance in accuracy of predicted results.

The data model stores historical data and reflects current state. This part accumulates relative data prepared for the construction of reaction rules on future uncertainties. In this part, by using methods like functional brain mapping analysis technology (i.e., f-MRI) or cognitive neurology analysis can obtain statistical characteristics of users' emotional and behavioral responses to situational context.

The feedback model is used to reflect response rules on uncertainties of future; this part will simulate the actual environment and predicts the following behavior if possible, so as to seek for effective solution or provide a basis for improvement to current solution in real-life situations. In order to reduce differences, the feedback system will begin a new round of optimization and evaluation procedure and generate error signal to further revise the assessment methods or parameters of artificial system through the observation of related state changes under the actual changes automatically.

**Figure 5:**
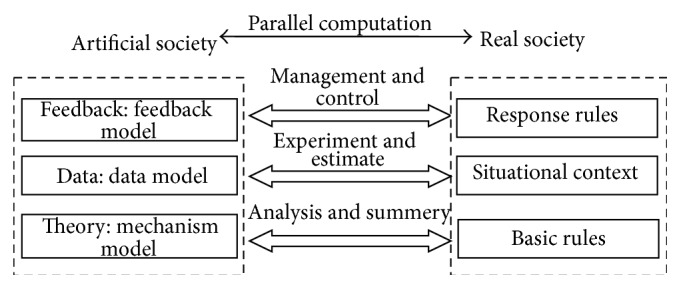
TDF framework.

By applying interactive iteration procedure and parallel computation analysis in both artificial and real society can obtain much better approximation descriptions of real environment and, in part, can predict the trend which in turn effectively manage and control changing problem in real social system. In this way, the model will be of great help to decision making for coping with dynamically changing situation.  Figure 5 shows the TDF framework for intelligent computation on social networks' information dissemination of public health emergency.

The application and realizing process of TDF framework can be divided into the following three steps.

*(1) Theory: Mechanism Model*. The famous disease spread model firstly presented by Grassberger in 1983 [31] has been widely used to describe the essential features of information dissemination in social networks. In this model, each node in a social network has two states *S* and *I*. Here, *S* represents the initial state, when each node receives a message; it can be transformed into *I* with certain probability. After spreading a message, node in state *I* will return to state *S* which has the possibility to be infected or delete the message that may forget or not be interested with the topic with certain probability. Consider new comers and inactive ID users; the dynamic transmission model can be expressed as follows [32]:

(5) $$\begin{array}{c} \frac{dS_{k}\left(t\right)}{dt}=b\left[1-S_{k}\left(t\right)-I_{k}\left(t\right)\right]-\lambda kS_{k}\left(t\right)\Theta \left(t\right)+\mu I_{k}\left(t\right)-dS_{k}\left(t\right),\\ \frac{dI_{k}\left(t\right)}{dt}=\lambda kS_{k}\left(t\right)\Theta \left(t\right)-\mu I_{k}\left(t\right)-\varepsilon I_{k}\left(t\right), \end{array}$$

where Θ(*t*) = ∑(*ip*(*i*)*I*_*i*_(*t*)/∑*kp*(*k*)).

It represents the probability that a given side connects with an information received node. Here *k* represents the degree of that node, *λ* represents the transmission threshold of a social network, *t* is a unit time, and *p*(*k*) is a distribution function of *k*.

In order to endow the prior knowledge and basic rules of mechanism model into the agents' simulation system in artificial society, the key attributes of agents can be designed as in Table 1.

Hereafter, the basic rules of mechanism model can be designed as follows.

When receiving a message, the changes in attributes of an agent are decided by

(6) $$\begin{array}{c} \text{Agent}\left(t+\Delta t\right)=I\left(Sd\left(a\right),At\left(T\right),Ad\left(T\right),E\left(t\right),p_{r}\left(t\right)\right). \end{array}$$

Here, *Sd*(*a*) is the attributes of the agent who sent that message, At(*T*) is the attention to this topic by the information received agent, Ad(*T*) denotes the attitude to the event reflected in the information which is initially fixed by interest, *E*(*t*) is emotion at time *t*, and *p*_*r*_(*t*) is the probability from state *S* to state *I*.

After sending a message, the changes in attributes of that agent are decided by

(7) $$\begin{array}{c} \text{Agent}\left(t+\Delta t\right)=O\left(E\left(t\right),p_{s}\left(t\right)\right). \end{array}$$

Here, we mainly consider the changes of emotions which may be decayed as *E*(*t* + Δ*t*) = *E*(*t*)*∗e* ^−*k*/Δ*t*^ according to the dynamic characteristics of emotions [6]. *p*_*s*_(*t*) is the probability returning from state *I* to state *S*.

The dissemination directions and scopes are decided by the social relationship Sr(*n*) of the information sending agent. Detailed relationships and parameters of formulas (6) and (7) will be achieved from data model by machine learning [32].

**Figure 6:**
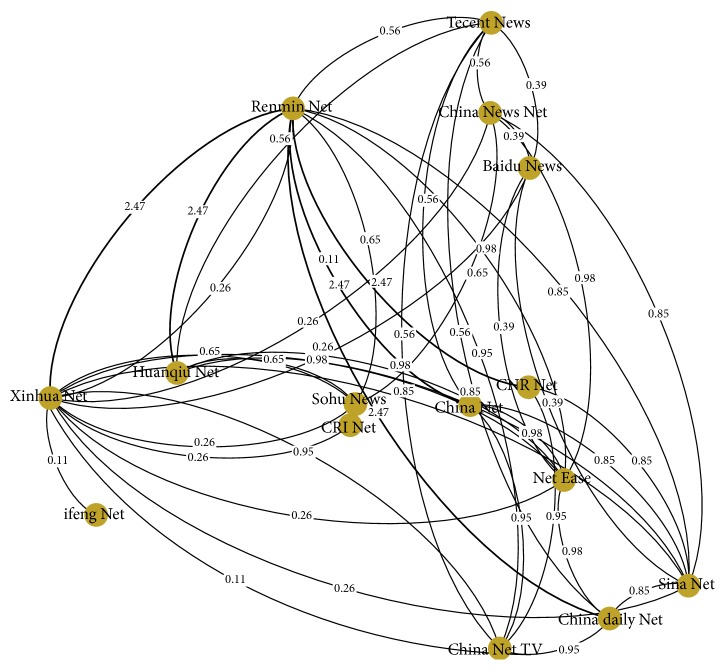
Relationship diagram of interconnections and strengths among websites.

*(2) Data: Data Model*. Data model should be built from the historical data under specific situational context in the real society. We selected mainstream media websites covering the total number ranked top 15 that were released by Chinese Internet data platform. These sites represent the vast majority of Internet users' access channel to news in China. We use these 15 web sites as nodes to generate web site correlation model by analyzing the link number of each site that pointed to other sites by weighted graph. With this model, we may track information about Internet users' personal browsing behavior affected from the strength of interconnection network sites, which will further affect netizens' emotions and cognition. Considering the information dissemination of public health emergency, we can obtain the relationship diagram as shown in Figure 6.

In Figure 6, the size of the node indicates the number of Internet users, and the weight reveals the strength between each node. The attributes of agents contained in each node can be endowed by machine learning from historical data. Technologies such as computations of attentions, attitudes, emotions, and information dissemination roles have been well developed in the existing researches [6, 14, 32, 33]. If only considering the macro information dissemination among websites, we can regard each website as an agent and endow their statistical attributes by machine learning [32, 33].

*(3) Feedback: Feedback Model*. As discussed in this paper before, the aim of ACP simulation system is to establish an artificial society which can “follow” the changes in the real society and predict its trends in the future by the parallel computation and interactive iteration procedure. Therefore, the changed information in the real society should be returned into the artificial society so as to adjust the attribute parameters of intelligent agents.

We designed a feedback system which can acquire and track the new information disseminations in social networks in the real society. The Cyber Psychological computation method [34, 35] is employed to evaluate the real changes in attentions, attitudes, and emotions of the member in a social network. Corrections of the agents' attributes are usually made every one hour. The predictions of their changes in the artificial society are based on the Kalman Filtering model as shown in formula (4). Besides, an online survey is also utilized to update the new response rules of people's possible behaviors under the upcoming situations, which can be combined into the rules of agents' activities in the artificial society. Generally, the analyses and predictions of information disseminations in social networks are based on the dynamic changes of agents' attributes as the results of their interactive activities in the artificial society, which is different from the traditional methods based on the computation of a mathematical prediction model.

## 4. Experiment and Results

Compared with the traditional methods, ACP simulation and TDF parallel computation have the superiorities of the feedback adjustment ability which can follow the changes in the real society and the complex description ability which considers the inherent psychological and behavioral mechanisms of the participants in information disseminations. Therefore, the proposed method can achieve an ideal prediction result and has been successfully applied in the analysis of complex public health emergency such as Bird Flu, A (H1N1) Flu, and Ebola Outbreak [6, 32, 34, 36].

Table 2 shows the dynamic information dissemination in social networks, which was recorded from 16008 nodes when an A (H1N1) Flu emergency took place in China in 2009.

**Figure 7:**
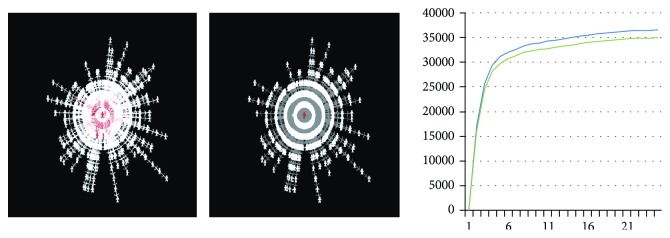
Information dissemination and prediction.

Figure 7 illustrates the information dissemination in social networks and the prediction of total number of disseminators. The left images in Figure 7 are the topology network of dissemination in social network within 24 hours. We point out the leading nodes (red nodes) and segment spreading groups into different strengths in this process. The right half in Figure 7 is the total number of disseminators. The blue line is the data of predication, and the green line is the actual number; it shows that the data of predication is very close to the real number. This indicated that the computation based on TDF framework can reach a very high precise prediction for the dynamic information dissemination in social networks. This method has been successfully applied to the national simulation platform for emergency management in China [32, 36].

## 5. Conclusion and Discussion

This paper analyzed the complex characteristics of information dissemination in public health emergency of social network as well as its network structure, cognitive psychology, and group behaviors and argued that the existing theoretical tools and modeling methods are not sufficient to accurately describe and predict the information dissemination in social networks. Therefore, a new intelligent computation method based on the framework of TDF (Theory-Data-Feedback) was constructed for the ACP simulation and prediction on the dynamic dissemination of emergency event's information and reached a high precise result.

## Figures and Tables

**Table 1:** Attributes of agents in artificial society.

Attributes	Parameters
Serial number	*n* = {1,2, 3,…, *N*}

Social relationship	Sr(*n*) = [0,1]

Attention to this topic	At(*T*) = [0,1]

Attitude	Ad(*T*) = [−5,5]

Emotion	*E*(*t*) = [−5,5]

Information dissemination role	(1) Opinion leader (2) Follower (3) Controller (4) The rest

Information dissemination state	DS = {*S*(*t*), *I*(*t*), *p*(*t*)}

**Table 2:** Information dissemination on social networks.

Time (hours)	Number of information dissemination
1	17107
2	8530
3	3896
4	1624
5	937
6	585
7	698
8	337
9	255
10	314
11	264
12	250
13	261
14	272
15	204
16	200
17	181
18	166
19	132
20	98
21	98
22	70
23	63
24	80
